# The immune system

**DOI:** 10.1042/EBC20160017

**Published:** 2016-10-26

**Authors:** Lindsay B. Nicholson

**Affiliations:** Cellular and Molecular Medicine, University of Bristol, Bristol, U.K.

**Keywords:** basic, immunology, review

## Abstract

All organisms are connected in a complex web of relationships. Although many of these are benign, not all are, and everything alive devotes significant resources to identifying and neutralizing threats from other species. From bacteria through to primates, the presence of some kind of effective immune system has gone hand in hand with evolutionary success. This article focuses on mammalian immunity, the challenges that it faces, the mechanisms by which these are addressed, and the consequences that arise when it malfunctions.

“*Great fleas have little fleas upon their backs to bite 'em,* *And little fleas have lesser fleas, and so* ad infinitum*.*” Augustus De Morgan (1872)

## Introduction

The problems that the mammalian immune system solves are not restricted to higher animals; they are faced by all forms of life and are ignored by none. The pressure that natural selection exerts is inexhaustible and unending. Emerging infectious diseases have as much potential to shape future human history as the epidemics and pandemics of the past. Managing this threat depends on understanding how to maximize the potential of our sophisticated immune system in the service of human health.

It is a fundamental property of immunity that no part of our body is cut off from its surveillance. For this reason, although the immune system may seem a less substantial thing than an organ such as the heart or the liver, in aggregate, immunity consumes enormous resources, producing the large number of cells that it depends on for successful functioning. After early childhood, most immune cells are produced from the **bone marrow**. Some of these then undergo very significant secondary education before they are released to patrol the body. Many important immune cell types have been identified. In a routine blood test, five different kinds of white blood cell will be counted (Table 1). An immunologist or a haematologist may subdivide these populations further, on the basis of the **proteins** that are expressed in their **cell membranes**. Among these proteins are **receptors** by which cells interact with each other and the environment. Receptors bind **ligands** which may be receptors on other cells, or soluble molecules such as **cytokines**. Cells express hundreds of different types of receptor on their surface. Many carry out fundamental functions, such as transporting glucose into the cell. The receptors associated with the immune system are generally concerned with interrogating the environment for evidence of danger, infection or abnormal cell death. In the course of an immune response, cells follow a programme, such that the overall outcome maximizes the likelihood of surviving and eliminating infection or cancer.

**Table 1.** White blood cells analysed by routine blood tests. Neutrophils and macrophages respond quickly to local infection; lymphocytes co-ordinate the adaptive response

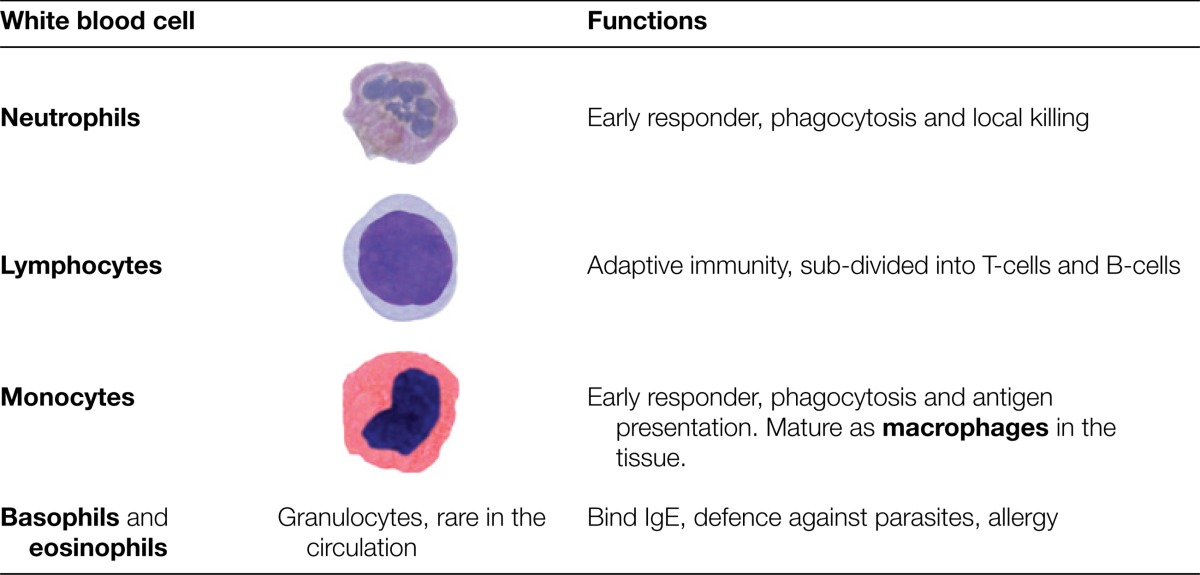

Receptors are also present inside the cell where they play an important role, acting to detect evidence of infection. Organisms such as **viruses** can spend most of their life hidden in the complicated **cytoplasm** of the cell, making them difficult to recognize from the outside. Receptors within the cytoplasm can bind to virus-derived signature molecules, such as different types of **nucleic acid** and signal that infection is present. Cells use a sophisticated system for sampling the proteins they are making, to check that none have come from viruses. If cells detect such telltale signs they respond, by producing cytokines that serve as alarm signals for surrounding tissues, and by committing rapid and effective suicide that leaves a cell remnant that can initiate adaptive immunity directed at the inciting infection.

Tackling infections is the job of different types of white blood cell. Early in an immune response, the most important of these are the **innate immune system** cells **neutrophils** and **macrophages**, which are the first at the scene of an environmental breach, such as an insect bite. Both of these cell types are effective killers in their own right. They secrete highly destructive substances including **enzymes** that digest proteins and reactive chemicals such as bleach that kill. Then they engulf and digest what they have damaged, a process called **phagocytosis**. Infections that are not destroyed by this attack attract the attention of **lymphocytes**. These cells embody the functions of adaptation and memory, allowing the immune system to make increasingly specific responses and to remember individual types of infection, so that reinfection is met with a faster and more effective counterattack.

All of these different responses rely on the selective expression of specific families of **genes**. Studies of the immune system have been at the forefront of characterizing how different gene programmes function. Immune cells read the environment through their receptors and then modify how they use the genes encoded by their **DNA**. Some groups of genes are switched on, and others are switched off. This gives the different cell types a great deal of flexibility in how they handle an infection. Sometimes these gene programmes change the cytokines that cells secrete, sometimes they change the pattern of receptors on the surface and sometimes they change how resistant the cell is to infection. Information in the environment can label a specific location, keeping immune cells from moving away. Other signals affect the whole body, such as the cytokines that stimulate changes in the regulation of body temperature that lead to fever. The adaptations that we make in response to infection are measured over many time scales. They may occur rapidly in minutes and resolve just as fast; they may continue for days until a viral infection is cleared or they may be long-lasting and change the local anatomy of a tissue such as the **autoimmune disease rheumatoid arthritis**. The immune system is, then, a highly connected web of many different types of response deployed to maintain the status quo of a **pathogen**-free internal environment.

### Challenges

An effective immune system must be able to interpret changes in the world around it and respond appropriately. To do this, it has to solve a number of specific problems.

#### Discrimination

Immune systems have an uneasy relationship with the environment. Most of the time an encounter with something new is harmless, but the small fraction of times that it is not can be very dangerous indeed. An effective immune system must be able to discriminate such differences, distinguishing self from non-self and distinguishing harmless non-self from dangerous non-self. For much of the 20th Century, research in immunology was focused on understanding how it achieved the former. It was spurred by an important early observation: that it was possible for animals to develop specific immune reactions against chemicals such as dyes, which had never existed in Nature. The ability to learn how to recognize these previously unknown structures implied that the immune system had solved the problem of how to classify and recall the shapes of individual molecules. Unravelling the biological machinery that achieves this was a signature achievement of 20th Century immunology.

This remarkable flexibility underlines the fact that the immune system interrogates the fundamental building blocks of the environment. A process of recognition at a near molecular scale allows the immune system to exploit the fact that all organisms are defined by proteins encoded in their genes. A **measles virus** is made of different proteins from those of a **rabies virus**. An ***Escherichia coli*** bacterium has a different structure from that of a **spirochaete**. By classifying the environment in terms of the proteins it contains, and by continuously sampling these proteins, the immune system executes a very active form of monitoring that it links to a stringent verification process and which must incorporate an ability to learn. As part of a defence against a potentially dangerous environment, each individual develops their own unique immune system, which acknowledges only itself. Everything that is not recognized might be a threat.

#### Flexibility

The immune system's ability to adapt flexibly to strange environmental changes is critical in fighting infections and cancer. Because our bodies have a remarkable capacity for renewal, and almost every cell is a factory working day and night to turn over worn out molecules, breaking them down into building blocks that are reused to make replacements, infection or cancer can arise at any time. Every time a cell divides, there is a small chance that it may develop a random unpredictable mutation that will transform it into a cancer. Infections reproduce much more rapidly than their hosts and can change their appearance to allow them to evade recognition. An effective immune system must cope with this unpredictability.

We can picture this as an ongoing evolution of the environment and it presents a special challenge for an immune system. In contrast with most organs, such as the heart, which does the same job throughout life, the immune system needs to adapt to an environment that is always changing. This problem is solved by investing in strategies that exploit the power of random change itself. Using randomness in this way creates waste, but preserves responsiveness. Even identical twins, which share the same genes, have immune systems that become increasingly different from each other from birth to old age, as each twin independently makes hundreds of thousands of unique random responses to the environment.

#### Managing infection

For a microbial infection to develop, the pathogen must get close enough to interact with individual cells. The skin and mucous membranes make this close approach difficult. Physical barriers provide innate protection, such as the tough overlapping cells of the skin and chemical barriers, and enzymes, such as **lysozyme** in snot and tears and the acid in the stomach, also kill many bacteria. These outward-facing surfaces actually encourage the presence of non-pathogenic microbes. By welcoming and supporting a co-operating microbial population, little opportunity is left for more dangerous relatives to move in. The healthy immune system lives happily with this symbiotic microbial farm, but still reacts when there is a dangerous infection. As our understanding of the ecology of this ‘**microbiome**’ grows, it may offer new therapies that can support the exclusion of disease causing organisms.

When pathogens do penetrate these defences and seek to live within our bodies and within our cells, they pose many threats, from quiet coexistence to wholesale cell destruction and death. There is wide diversity in pathogens’ methods of attachment and entry. For every individual pathogen, this process is tailored to species, to specific cell types and to defined cell-surface receptors. Each infection uses a different door into the cell. Blocking off these routes of entry can stop an infection before it starts. By producing **antibodies**, the immune system can neutralize an infection before the key to the cell turns in that particular doorway. But this must be carried out one key at a time.

A pathogen that has penetrated the defences of the skin and mucous membranes and established itself within or between cells, or a cell that has turned into a cancer, can only be eliminated by killing. This is a dangerous business, and when the immune system is battling with an infection, it may put the life of the host at risk. Sometimes when it is not infection, but an adverse reaction to a drug or a treatment for cancer which activates the immune system, this leads to critical illness. There is a delicate balance between what is successful and what is sustainable when invoking a full-blown immune reaction.

Moreover, some infections cannot be killed off reliably by the immune system. Viruses that evade immunity, by hiding within cells, lead to repeated bouts of illness as limited as cold sores or as destructive as AIDS. Cancers that escape from immune control can continue to grow, may metastasize and then kill in different ways.

In meeting all of these kinds of assault, there is a middle path that an immune response must keep to, between too much destruction and not enough. In this zone, it is quite ruthless in sacrificing itself to terminate an infection. When common cold viruses have hijacked cells in the throat to replicate, these virus factories are not rehabilitated, but destroyed by killer cells. Where a bacterium has penetrated the skin and established a viable colony, suicidal white blood cells fill the area with bleach that they make themselves, killing indiscriminately and leaving behind a wasteland of debris that needs to be engulfed, digested and processed by the immune system.

#### Memory

One of the most significant features of the immune response is its ability to retain a memory of previous infections. This both protects individuals from reinfection and limits the spread of infection in a community. Immune memory can be very long-lasting; when adults were studied, their memory for the measles infection was decaying so slowly, it would have taken over 3000 years to decrease by half. This goes well beyond life-long protection. These robust durable changes are the reason that, when we vaccinate, the protection this produces delivers long-term benefits.

Within an individual, immune memory must be distributed throughout the body. Circulating antibodies travel in the blood, reaching everywhere the circulation does; memory also develops outside the bloodstream within tissues. Killer cells can remain on guard where the immune defences broke down in the past, alert but not activated, ready to attack rapidly if reinfection occurs.

Finally, some infections have such a profound impact on a species that the imprint of individual pathogens can be perceived in the tree of evolution. If an infection is lethal, only individuals who have genes that encode effective resistance will survive to produce the next generation. Modern methods of analysing inheritance have demonstrated how the co-evolution of host and infection has shaped the make-up of the immune system and the receptors it uses for recognizing and fighting pathogens.

All immune systems address and solve these challenges. How mammals achieve this complex task is the story of an integrated system of biological processes, often using strategies that surprised their discoverers, whose elegance and power continue to provide new insights for students of immunology young and old.

## Discrimination

### Adaptive immunity

**Figure 1:**
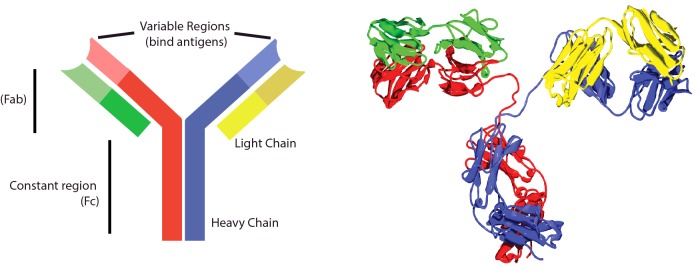
Antibody structure Antibody cartoons are often drawn in a Y-shape, as on the left. This picture represents an IgG molecule, made from two identical heavy (red and blue) and two identical light (yellow and green) chains. The top of the Y (called the Fab region) contains two variable regions that each bind the same antigen. The bottom of the Y is the constant (Fc) region, which interacts with cell receptors, complement, etc. On the right is a crystal structure of an IgG2 antibody. The heavy chains (red and yellow) and light chains (blue and green) are identical. This antibody is ∼10 nm from bottom to top. (From TimVickers. https://commons.wikimedia.org/wiki/File:Antibody_IgG2.png)

The immune system uses many different receptors to interrogate the environment. These are usually proteins and are found in the blood, in tissue fluids or bound to the cell surface. The antibody receptor, also called an immunoglobulin (Ig), was the first **antigen**-specific receptor to be characterized and is commonly drawn as a Y-shaped cartoon. It is formed by the combination of two identical heavy and two identical light chains. Such an Ig comprises three globular domains connected by more flexible linkers; the two binding domains, coded for by variable regions, have identical specificity for antigen (Figure 1). The globular Ig structure is a widely adapted template that is used by many molecules both inside and outside the immune system. The structure is known as the Ig domain (or fold) and proteins that contain such a domain are members of the ‘**immunoglobulin superfamily**’.

**Figure 2:**
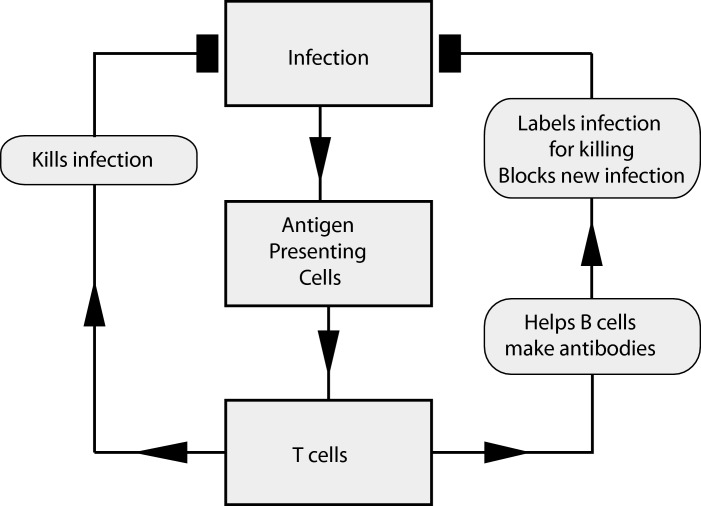
The adaptive immune response to infection Infection, detected by APCs, triggers specific T-cells that co-ordinate killing and antibody production which stop the infection.

Antibodies are soluble and do two things. At one end, they bind firmly to a target (antigen), whereas at the other, they signal to immune cells. Antibodies are divided into a number of different families, called **isotypes** (Table 2) and their production is a carefully regulated process involving cell–cell interactions that control which antibodies are made. Antibody research in the first half of the 20th Century focused on antibodies that could be purified from **serum**. Once the **amino acids** that made up the different chains were defined, attention turned to understanding how antibodies were produced by individual cells. It was discovered that, for most antibodies, their generation depended on co-operation between at least two types of cell: a cell that processed and presented targets (antigens) from the environment [**antigen-presenting cell (APC)]** and a lymphocyte that recognized the target antigen on the APC. This lymphocyte (now called a **T-cell** or T-lymphocyte) directed either the production of antibodies or killed the cell presenting the antigen (Figure 2).

**Table 2.** The five main types of antibody isotype which have different roles in the immune response

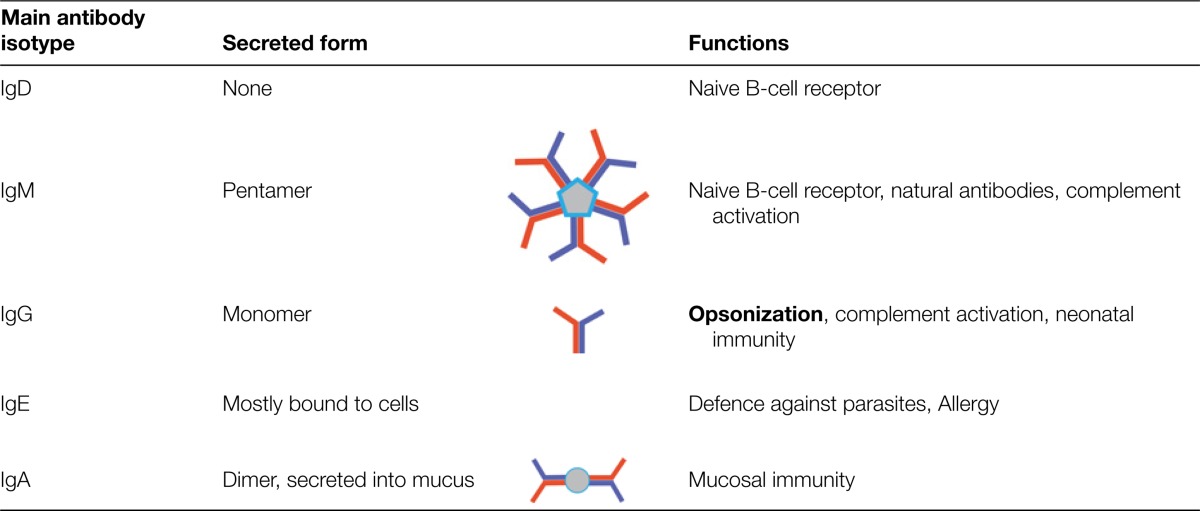

One early idea to explain how T-cells determined what to respond to was that the immune system only presented antigens from infections, but this was wrong. To the surprise of many immunologists, studies in the 1980s which defined the receptor molecules on the surface of cells that controlled this targeting process revealed that essentially all cells presented antigens. A healthy immune system surveys these antigens constantly, but this does not provoke a response. This challenges the impression that the immune system spends most of its time doing nothing. Quite the opposite: it is constantly reviewing the environment, checking whether anything is amiss. The immune system has a carefully developed sense of self which it generates through a process of education.

**Figure 3:**
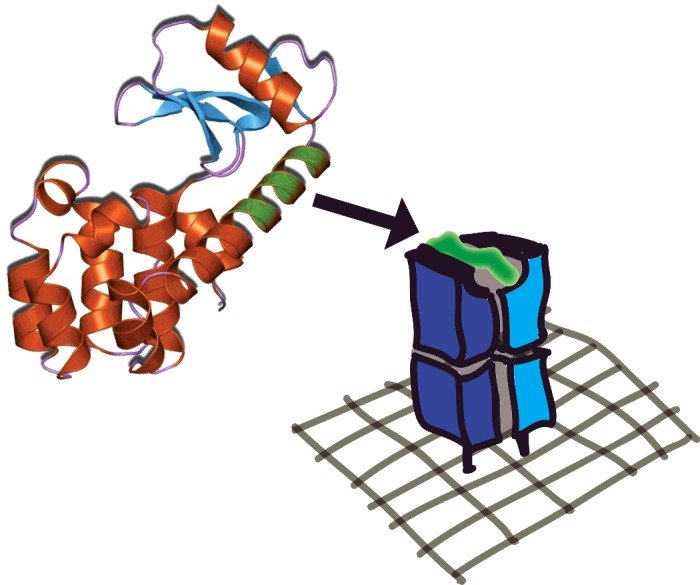
Antigen processing I A protein molecule (on the left) is digested by the cell and a fragment from it (shown in green) is loaded into an MHC molecule that is then displayed on the surface of the cell (on the right), oriented so that it can be scanned by T-cells. The MHC molecule is ∼7 nm tall above the cell membrane. Not to scale. Jawahar Swaminathan and MSD staff at the European Bioinformatics Institute https://commons.wikimedia.org/w/index.php?curid=6292018

The lymphocyte's antigen receptors recognize a family of cell-surface molecules on APCs that are collectively known as **major histocompatibility** (MHC) determinants. In humans, these are also called **human leucocyte antigens** (**HLA**s). These molecules are a combination of material derived from the environment (the antigen), bound in the flexible jaws of the MHC molecule which hold it in place. The rest of the MHC molecule acts as a scaffold orientating the MHC–antigen complexes at a cell's surface where they can be scanned by a lymphocyte (Figure 3). In this way, cells display a constantly changing picture of the proteins that they are making as antigens loaded into their MHC molecules, and these antigens come from inside and around cells. This sampling strategy is effective because disguise is very difficult at the molecular level. A bacterial cell makes many proteins that do not resemble those made by an animal cell; a cell making a virus inside does not look like a normal cell.

**Figure 4:**
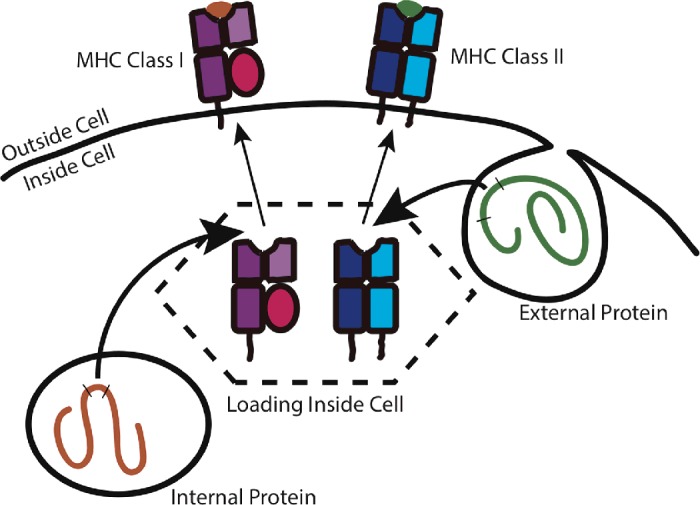
Antigen processing II Internally produced and externally captured proteins are loaded on to MHC molecules inside APCs. Internally produced proteins are presented by MHC class I molecules, which are found on all the cells in the body. Externally acquired proteins are presented by MHC class II molecules on specialized APCs. Once loaded, molecules are exported to the cell surface.

The commonest type of antigens presented by MHC molecules are **peptides,** short stretches of amino acids (eight to 30) which are processed from proteins. The source of this material may be the internal or external environment of the cell that presents it. Two types of MHC molecule, class I and class II, are involved in this process. All cells, except for red blood cells, present a selection of antigens from the proteins that they are making bound by MHC I. Each cell wears a collection of MHC–antigens on its surface that act as bar codes, identifying that cell to the immune system. Some immune cells called APCs are specialized to present antigen, and load antigens into MHC I and MHC II. They are continuously hoovering up proteins from the environment, using enzymes to digest them to produce short peptides, and loading them into the jaws of the MHC within the cell, before these molecules are displayed on the surface. In APCs, these two parallel processing pathways provide a real-time survey of whatever the environment contains (Figure 4).

All MHC molecules work in the same basic way, as a scaffold to present antigen, but as a species we have in our heritage a repository of many different MHC molecules (1122 common and well-documented different **alleles** as of 2013). This diversity at the level of the population of all humans, is narrowed down in the individual, who inherits about nine MHC molecules at random from their parents. Your own MHC molecules define your **tissue type** which is the major barrier to successful organ transplantation, and the reason we keep records of the tissue type of potential donors. The chance of meeting a stranger who shares the same MHC molecules as you is tiny, which is why, among a panel of 20 million potential donors, 2–5% of individuals will not find an exact match. Even an individual with the commonest set of HLA genes found in the U.K., who needed a transplant, would only find a few hundred potential donors among this panel. This is also why your best chance of finding a compatible donor is searching among your close relatives, with whom you share genes.

Of course, the complexity of this system did not arise to frustrate transplant surgeons. The immune system uses it, because each of these many different MHC molecules presents a unique selection of antigens processed from the same underlying proteins. For example, the antigens that are presented from the liver of one person will be different from the antigens presented from the liver of an unrelated person. In this way, the representation of self established by an individual's MHC, presenting its self-proteins to its own lymphocytes, is a very private system of identification that is difficult to copy, allowing the immune system to discriminate between foreign tissue transplants, invading infections and cancerous cells.

**Figure 5:**
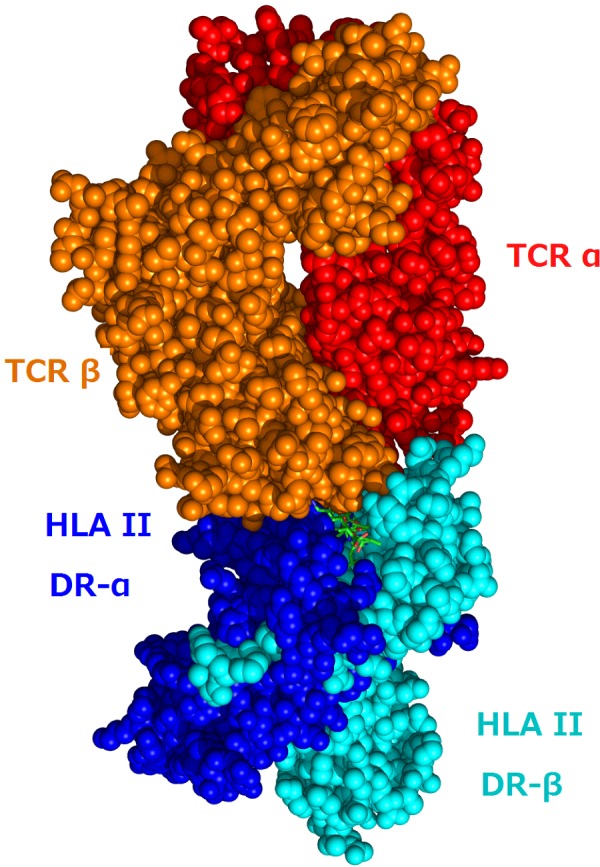
MHC–TCR interaction Crystal structure of the interaction between a TCR (the α and β chains) and human MHC (HLA DR α and β chains). The peptide antigen can just be seen at the interface between the two receptors. Influenza peptide, class II presentation https://commons.wikimedia.org/wiki/File:1FYT_T-cell_receptor_and_HLA_class_II_complex.png

The sensor that interacts with the MHC–antigen complex is called the **T-cell receptor** (TCR or TcR). This receptor is part of a complicated structure called the **TCR complex** (it is formed by the combination of six different protein chains in four pairs). Two TCR chains (α and β) make up the sensor that examines MHC molecules (Figure 5), and the other chains are used for signalling the results of that examination into the T-cell. They are often omitted from cartoons that show the TCR at the cell surface. The signals that the TCR generates within the T-cell depend on the **affinity** of the interaction of the α and β sensor chains with the MHC–antigen. In essence, each TCR measures the affinity of this interaction and provides a read-out to the T-cell which determines the subsequent response of that cell. An activated TCR sends signals across the cytoplasm of the cell to the **nucleus** where it initiates programmes that change the pattern of genes that are being expressed and therefore some of the proteins made by the cell.

Complementing the wide variety of possible MHC molecules, TCRs need a comparable level of diversity to ensure the availability of suitable receptors. This is achieved through a modular approach to building up the receptor. When a new TCR is being made, parts of the α chain and β chain, which are used for recognition, are generated at random from a pool of hundreds of gene segments. These are joined together in a process that also adds random mutation. This means that at any time there are many more possible ways and individual can make TCRs that there are T-cells in the body. Each T-cell carries multiple copies of a single unique TCR.

**Figure 6:**
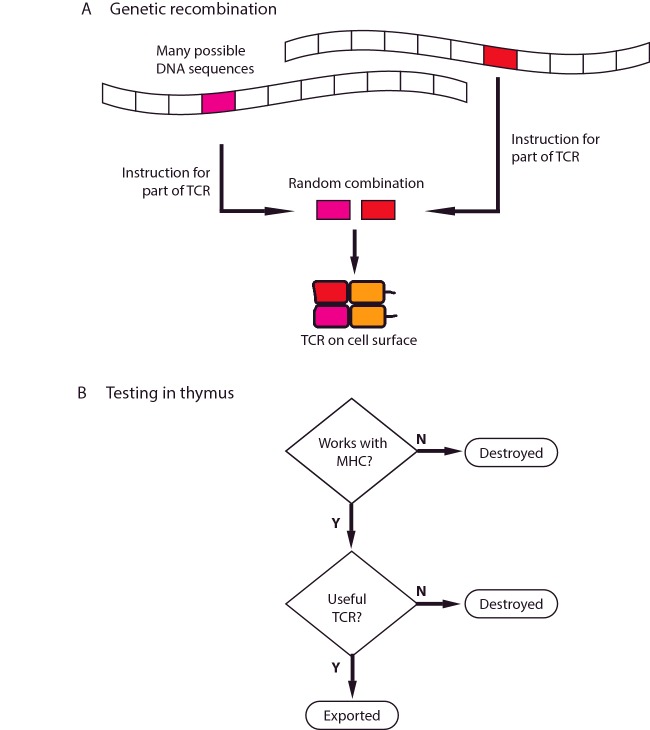
TCR and T-cell selection (**A**) The DNA encodes a large number of different possible sequences for the TCR. The code for the part of the TCR that recognizes antigen are selected at random from these sequences. Different structural elements are combined to make the final TCR. The resulting receptor is expressed at the cell surface and tested in the thymus. (**B**) Cells bearing each TCR are put through a number of screening tests. If the TCR cannot work with the individual's MHC molecules, or if it is useless or dangerous, it is destroyed. If it passes these tests, it is exported from the thymus.

Because making a TCR involves random recombination, this is quite a wasteful process, and only a small fraction of the T-cells produced will have TCRs with optimal biochemical properties. To test for these characteristics, T-cells first pass through a specialized organ called the **thymus** (which gives T-cells their name, T for thymus). For the T-cell to survive selection, the TCR must be useful. It must be able to ignore **self-antigens** in MHC complexes, but must still bind the MHC molecule strongly enough to interact with the peptide antigen. If this binding is too weak, the cell is discarded. TCRs that produce strong activation by self-antigens are dangerous and they are also removed by screening (Figure 6). To match these stringent requirements, the immune system is prepared to throw away more than 19 out of 20 of the T-cells that it makes. The result is a population of T-cells with a repertoire of TCRs that recognize self-antigen weakly, have the potential to recognize non-self-antigen strongly, and can safely be exported from the thymus.

In a healthy person, these exported T-cells move continuously between **lymph nodes** and the blood, testing APCs for signs of infection. This baseline surveillance requires that the T-cells actively engage with APCs, using their TCR to interrogate MHC–peptide receptors. Usually they do not encounter activation signals and they move on. After several weeks, they will be replaced by younger cells, newly exported from the thymus, carrying their own unique TCRs.

But when an individual develops an infection, and antigens from this infection begin to be displayed on APCs that are scanned by T-cells, a few of the millions of different T-cells will have TCRs that trigger activation of the T-cell. First this stimulates the cell to divide, producing daughter cells with the same TCR. Instead of the blood only holding a handful of infection-specific T-cells, this expansion leads to it having first thousands and then millions. These cells also acquire an ability to act (Figure 2). **CD8**^+^ T-cells may kill infected cells directly, and **CD4**^+^ T-cells help to make antibodies; both send out signals to attract macrophages and neutrophils to the site of the infection. As the infection progresses, responding cells become more specialized, developing different **effector functions** that optimize how the immune system attacks it.

The other crucial recognition system of the adaptive immune response, antibodies that are produced by **B-cells**, go through a process of maturation and selection that serves to greatly increase the strength with which they bind their target antigen. Antibodies are very different from T-cells because, although they start as a cell-surface receptor [the **B-cell receptor** (**BCR**)], they are later secreted and can function well in many places that T-cells do not. Once they have been produced, they are an efficient early defence in cases of reinfection, able to bind to pathogens that breach external barriers. They can also neutralize soluble poisons (**toxins**) that some organisms produce, which is very important, for example, in the response to diphtheria. Antibodies circulate in the blood, are found within the mucus that lines our gastrointestinal organs and also in interstitial tissue fluids. Mothers pass antibodies to their children through their breast milk.

**Figure 7:**
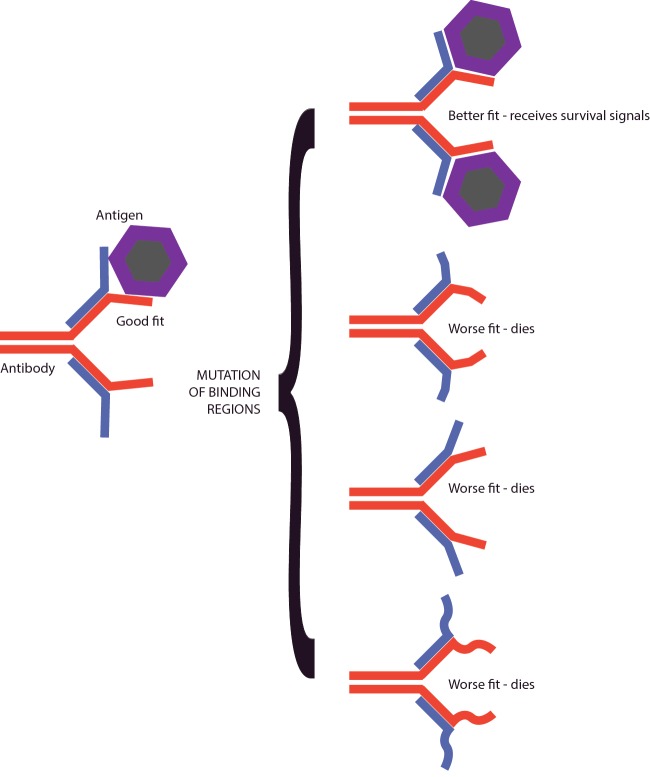
Affinity maturation Antibodies that fit quite well are selected early in the immune response. The antibody receptor (BCR) is mutated within daughter cells. Many of these mutations bind the antigen worse than the parent antibody, and cells producing these antibodies die. Some antibodies bind the antigen better than the parent, and these cells live.

Like TCRs, antibodies are adapted to specific infections by selection from a pool of randomly generated candidates, through a series of selection steps called **affinity maturation** that promote optimal function (Figure 7). But, unlike TCRs, antibodies can recognize whole proteins before they have been broken down into peptides. The majority of antibodies recognize proteins in their native state, folded up, with different chains and loops contributing to a patch on their surface to which the antibody binds. Because of this antibodies often focus on the outside of pathogens. And, as a defence, some pathogens, from the influenza virus to the malaria parasite, have developed processes that continuously change how they look at the surface, as a way to escape the immune system.

Antibody production starts in specialized immune system tissues. When a pathogen invades, antigens from it are carried to areas inside lymph nodes or the **spleen** by APCs. B-cells that have a BCR that can bind to these antigens are activated, take in and process antigen and load it into their MHC molecules. This prepares them to receive signals that stimulate growth and affinity maturation from T-cells that recognize these same antigens. B-cells and T-cells attract each other and this mutual attraction facilitates encounters between rare antigen-specific B- and T-cells. The help that T-cells provide to the B-cell promotes secretion of antibody, changes in the isotype of antibody that is secreted, and stimulates mutation of the genes that code for the BCR. Because the mutations occur at random, most of them actually impair the ability of the BCR to bind antigen, another expensive process in which the immune system is prepared to throw away many cells to select the best one. A fraction of the mutations improve the binding affinity of the BCR, making it stronger. Cells with BCRs of a higher affinity compete better for reducing amounts of antigen and are selected to live, whereas their less effective siblings die. By repeating this process several times, the affinity of the antibodies that are being made can increase by many orders of magnitude.

This dramatic increase in specificity is a key part of immune memory, but in recent years it has also become applied to many new ‘**biologics**’; therapies using antibodies to target diseases as diverse as rheumatoid arthritis and lung cancer. The high specificity that antibodies offer have made them an extremely flexible and effective medical technology.

Isotype switching permits antibodies to be directed into different roles. By rearranging the genes that specify the heavy chain of the antibody, Igs can be optimized for different environments and functions. The different isotypes are named according to their heavy chains (IgM, IgD, IgG, IgE and IgA). Because the antigen-recognizing domains of the antibodies do not change when a molecule with a new heavy chain is produced, each clone of B-cells maintains its specificity. A summary of the functions of different isotypes is given in Table 2.

### Innate immunity

Discrimination is also a key aspect of the **innate immune system**. This is an area of research that has grown explosively since studies at the close of the 20th Century identified families of receptors that sampled the environment for the presence of molecules associated with pathogens (called pathogen-associated molecular patterns; PAMPs). One of the first pathways of this kind to be worked out in detail exploited the discovery that a receptor cloned from insects, and implicated in their sensitivity to infection, was related to the gene for a similar protein that could be found in mammalian cells. This molecule, called **Toll-like receptor 4 (TLR4)** is a key mediator of the effects of **sepsis** in patients critically sick with infections. When it binds to bacteria, TLR4 triggers the release of cytokines that stimulate the whole immune system producing fever and, in the seriously ill, shock. TLR4 signalling can trigger the activation and recruitment of neutrophils and macrophages that can kill or limit the spread of infection at an early stage, allowing time for the adaptive immune response to develop.

Receptors that recognize PAMPs are found both outside and inside cells. As a group, these innate immune system receptors survey the environment for everything from viruses to fungal infections. The scope of this sensing network is an indication of the importance that an early reaction to infection plays in the survival of its victim. Innate immune responses slow infections down, giving the rest of the immune system time to catch up. Disorders of the innate immune response cause ‘**autoinflammatory’** diseases, often manifesting as spontaneous bouts of illness and fever.

The discrimination of appropriate targets that require an immune response from those that do not is the key to immunity. Unleashing the immune system is a risky business. If the reaction is too strong, it can kill its host. If it is not strong enough, the infection may do the same. To carry out this difficult balancing act, the immune system comes with many checks that operate as the response to a pathogen progresses. One of the first mechanisms is a process of double-checking, before even starting to respond, called ‘**co-stimulation’**. The need for co-stimulation was deduced once it was understood that the immune system could generate antigen-specific receptors at random and throughout life. By continuously making new and unique T-cells and B-cells, there is a constant risk of producing an **autoreactive** antigen-specific cell that targets self-antigens. To explain how receptors that recognize self-antigens could be turned off rather than on, if they bound a self-antigen, it was suggested that full activation of immunity required two signals, one from the antigen-specific receptor and a second from a hypothetical co-stimulatory pathway. This insight, developed ahead of the description of the receptor pathways by which it operated, was very fruitful in explaining how antigen-specific signalling could have two apparently opposite effects, one shutting down the cell, the other spurring full activation. By adding a checking mechanism to the activation process, the immune system increases its discriminatory power, reducing the risk of errors. As these mechanisms became elucidated down to the level of individual signalling pathways, it was possible to uncover how this strategy has been applied to a number of different types of immune cell interaction. Another important check is provided by antigen-specific T-cells that shut down responses. These are called T regulatory cells. This population is devoted to negative feedback, limiting dangerous responses to autoantigens.

In summary, immune discrimination is distributed across many cell types. It depends on recognizing the molecular signatures of the diverse world of pathogens. To initiate a rapid first response, it employs many receptors that have become ‘educated’ through the process of evolution, to bind with high specificity to molecular structures associated with infection, and then signal to destructive cells like neutrophils and macrophages to attack (innate immunity). A second sophisticated system, which recognizes and ignores ‘self’, attacks antigens that it does not know (adaptive immunity). By using a highly variable family of MHC molecules that are essentially unique for each individual, every immune system sees the world of pathogens differently, making it difficult for infection to develop an effective disguise. By constant surveillance, the immune system provides a safe space to carry out normal functions of life. And, as a bonus, the immune system can carry on learning, so that long-lived animals that are exposed to new rapidly dividing infections, have recourse to real-time evolution of immune memory that can defeat infection.

## Selective killing

The most desirable immune response is one that stops an infection in its tracks, before it has established a foothold in the body. Phagocytosis of bacteria in the tissues and antibody-mediated blockade of virus entry into cells work this way. But if, once an infection was established within a cell, the immune system did no more, or if a cell that had turned into a cancer was ignored, viruses and cancers would be unstoppable. To deal with these eventualities, cells of the immune system control powerful lethal weapons. This ability is so striking that the cells that specialize in execution are known as **cytotoxic** killer cells.

Killers discriminate by using recognition receptors. Cytotoxic T-cells use the TCR and the CD8 co-receptor which together interact with MHC I. In this way, they can interrogate any nucleated cell in the body. When cytotoxic T-cells recognize an infected target cell, they kill it rapidly and move on to the next cell.

**Natural killer cells**, part of the innate response, use a different approach to selecting their targets. These cells patrol the body asking themselves whether the tissues that they survey express MHC I molecules. If the cells that are being examined do, then they move on, but if not, they will become activated and kill. This provides an alternative method of surveillance, which does not depend on specific antigen, and frustrates a strategy that some pathogens employ, of inhibiting the surface expression of MHC I. By insisting that nucleated cells report on their protein production, the window of opportunity that viruses have to squeeze through to be successful is narrowed further.

Cell killing is a specialized function that occurs in a series of steps. First the cytotoxic cells make close contact with the target and mobilizes intracellular granules to this area of contact. Then these granules fuse with the cell membrane of the cytotoxic cell and release a number of proteins. One, called **perforin**, forms a pore in the cell membrane of the target. This pore allows the entry of other proteins called **granzymes** that trigger rapid cell death. A second pathway, that probably plays a minor role in cytotoxicity, is that involving a receptor on the cytotoxic cells called **FasL** binding to its partner on the target cell called **Fas**. This interaction triggers a suicide signal within the target cell.

All cells are able to commit suicide and this type of cell death is called **apoptosis**; it is very important in development and in the immune system. In the thymus, where, as explained above, most of the lymphocytes that audition for a role as useful effector cells die, it is through the process of apoptosis that this occurs. Apoptosis also frustrates some viral infections and protects individual cells from becoming cancerous. Cancers only develop if they have mutations that block the activation of apoptosis, and viruses produce proteins to stop apoptosis switching on. In this way, the transformed or infected cell can survive signals that would otherwise lead to it killing itself.

The final common pathway of apoptosis is a **proteolytic cascade** which digests the contents of the cell and fragments its genetic material; the cell shrivels up and exposes signals at its surface that tell neighbouring phagocytes to eat it. The enzymes that carry out this process come from a family called **caspases** (cysteine proteases that cleave proteins after aspartic acid residues). There are several different ways to initiate this process, but they share a common final pathway. One feature of ‘normal’ apoptosis, such as occurs in the thymus, is that it does little to stimulate inflammation. This means that cells can die without initiating an effector immune response. In contrast, in an aggressive infection, where cells death occurs alongside signals that stimulate innate immune activation, accompanying adaptive responses will also occur. Successful immune responses reach an appropriate match between the threat and the response, producing enough killing to manage infection, but not so much that the host is compromised. If this is not achieved, the outcome may be disastrous in the short-term, because an infection is not controlled, or because the immune response is so aggressive that the body collapses. Or dangerous in the long-term because a chronic infection becomes established or because the immune system over-reacts and develops specific responses that attack healthy tissue.

## Managing infection

Developing precisely targeted adaptive immunity is a core capacity of the immune response, but there are many other important and interlocking effector functions which are essential to successfully overcoming colonization by a pathogen, or adapting to live with long-term infection. As our understanding of them continues to grow, we learn how to exploit these mechanisms to treat disease. But we can also look back at the beginning of immunology and see how many of the themes were established early in the history of the science.

In 1882, a Russian refugee called Elie Metchnikoff carried out an experiment on starfish larvae. He put small thorns into them and when he returned the next morning to study the result microscopically, he discovered that the thorns were surrounded by cells. This experiment changed the course of his research, alerting him to the importance of cellular function in immune responses. These cells, which were phagocytes, could ‘eat’ infections to protect the host. In subsequent decades, following this discovery, there was a fierce debate weighing up the relative importance of such cells. We now understand that they are a crucial element that functions alongside antibodies and **complement** activation fighting infection. Phagocytes are part of the first wave of response to many kinds of infection. Two types of cell, called neutrophils and monocytes, are produced and stored in reservoirs in the bone marrow. Early soluble signals, released into the blood in response to infection, stimulate increased cell production in the bone marrow and the release of phagocytes into the circulation, where they can be detected as a sign of infection by blood tests measuring white cell numbers. This early mobilization can slow down the spread of pathogens, trapping them locally and hindering their growth.

Macrophages and neutrophils also produce toxic chemicals that kill, and enzymes that disrupt tissue. For the individual cells, this is a suicidal strategy: they are sacrificed to end infection. This destruction can result in the formation of an abscess; a collection of dead immune cells and dead bacteria. Such a focus of infection can be relatively benign, if it is localized to peripheral soft tissues and heals quickly. The same kind of response in a more vulnerable tissue, such as the kidney or the eye, can cause irreversible damage. It may eliminate the infection, but destroy the function of the organ that has been affected. Healing that is delayed or incomplete, because the infection cannot be cleared, can produce chronic damage and long-term disability. Antibiotic therapy is some help, but chronic infection of this kind continues to be a cause of illness and disability worldwide.

Eating infections is not without risk for the phagocyte. Following engulfment, the target is surrounded in a structure (a **phagosome**) that fuses with a second organelle called a **lysosome,** which provides enzymes to digest the bacteria. These enzymes are activated by lowering the pH of the environment. Certain infections, such as tuberculosis (caused by *Mycobacterium tuberculosis*) and Legionnaire's disease (cause by *Legionella pneumophila*) resist this process, inhibit the fusion of the phagosome with the lysosome, and use this route as a way to hide from adaptive immune responses and to disseminate throughout the body. Infected macrophages can be stimulated to clear their load of *Mycobacterium,* but this needs help provided by antigen-specific T-cells that recognize the signs of tuberculosis infection in the MHC molecules and release **interferon γ**. Here the adaptive and innate immune responses can be seen to be co-operating to destroy an infection that is hidden within cells.

Adaptive immune responses can also be very important in interrupting the development of disease. It takes time to produce specific antibodies, because they only develop naturally during and after an episode of infectious disease. Getting the immune system to generate them, without suffering from the disease, can be brought about by **vaccination** as discussed below. If they are present, antibodies activate the immune system with speed and specificity. Their exceptional sensitivity facilitates a rapid and targeted response, which can halt an infection before it provokes any symptoms. Antibodies interrupt the transmission of viruses by binding to surface proteins needed for cell entry. The different strategies that viruses use to enter cells all depend on specific molecular recognition. Interfering with this specific binding process will stop the virus in its tracks. This kind of neutralization is also effective against protein toxins, such as the diphtheria toxin, that bind to a receptor at the cell surface as the first step in their attack. Specific antibody that binds the molecules needed for cell entry by a virus or by a toxin prevents disease developing.

Antibodies do not just interfere with entry into cells. Natural antibodies are all bifunctional (Figure 1). At one end is the structure that mediates specific recognition (called the **Fab fragment**), but at the other is a tail (called the **Fc fragment**) that engages directly with other elements of the immune system. This interaction can be a simple ‘eat me’ signal coating bacteria and attracting the attention of phagocytes, or a ‘kill me’ signal that recruits killer cells. Some Fc tails bind and activate complement protein that are present in the blood. This starts a powerful proteolytic cascade that forms pores on the surface of bacteria and punch through their tough cell walls, killing them and leaving them for disposal by phagocytes. Another type of Fc tail produces an isotype called IgE, attaches antibodies to the surface of **granulocyte** cells. If they encounter an antigen that they recognize they signal the granulocytes to release inflammatory mediators such as **histamines** and **leukotrienes**. These responses are especially relevant in immunity to parasites and in diseases such as asthma and allergy.

The Fc part of an antibody molecule is also used as a signal to transport it across cell barriers. The majority of new antibody that a healthy human produces ends up outside the body in the intestines and in the lungs. Once in these mucosal locations, such antibodies can bind and neutralize pathogens and toxins, before they have the opportunity to cross into the circulation. Because all antibodies facilitate specific recognition but have tails that deliver different functions, they are named on the basis of their tails (Table 2).

The path that immune responses follow does not always lead to the complete elimination of infection. Cold sores caused by herpesviruses and tuberculosis are two examples of pathogens that achieve long-term colonization in susceptible individuals. When we study antigen-specific immune cells in these circumstances, they appear to be very different from both naive cells and effector cells. Aggressive immune responses are shut down in two ways. The first is intrinsic to the cells that have been attacking the infection, and depends on the up-regulation of **co-inhibitory receptors** on the cell surface. Signals from these co-inhibitory receptors switch off attacking functions, such as cell killing, and terminate local destruction of tissue and of pathogen-infected cells. This is a trade-off between continuing damage and tolerating the presence of infected cells. The extrinsic type of regulation is the expansion of the antigen-specific T-regulatory cells, which secrete anti-inflammatory cytokines that limit immunity and promote tissue healing. Regulatory cells, which are stimulated by tissue antigens, play a very important role in managing the immune system. Mutations that cripple them cause severe autoimmune disease in animals and people, showing how crucial the control of immune responses is to health.

These regulating pathways are meant to be beneficial, but they can be exploited by disease. This is particularly significant in cancer. Tumours are commonly infiltrated by many different types of immune cell. But although these cells can often respond to cancer antigens, they do not succeed in killing the tumour, and this is because successful cancers produce signals that drive the immune system towards self-regulation, shutting off aggressive potentially tumour-destroying effector mechanisms.

Reviewing how the immune system manages an infection, there is a clear pattern. In the beginning, signals attract early non-specific cells that can kill and limit spread, then there is an explosive adaptive immune response that recruits antigen-specific cells and produces high-affinity antibodies that can block key molecular signals that the pathogen need to thrive. Finally, either there is a successful resolution that eliminates the danger or the immune cells negotiate a truce through a delicately adjusted process of regulation that preserves the function of the whole, even at the expense of tolerating ongoing colonization (see Appendix 1 for a summary).

## Memory

Memory is the signature attribute of the immune system. Individuals who recover from a specific pathogen resist reinfection with the same disease. This striking outcome is the key to vaccination, so it may be a surprise there is still a great deal to learn about how immunological memory develops. The benefits of an immune system that can fight off measles more effectively the second time it tries to attack is obvious. But the ability of the immune system to learn is also critical for a more subtle reason. One advantage that pathogens have over mammals is that they can evolve much faster than their hosts. An adaptive immune system that can learn to recognize a new infection in a few weeks, rather than having to wait a lifetime to develop an effective defence, provides a bulwark against emerging diseases that have never been encountered before.

**Figure 8:**
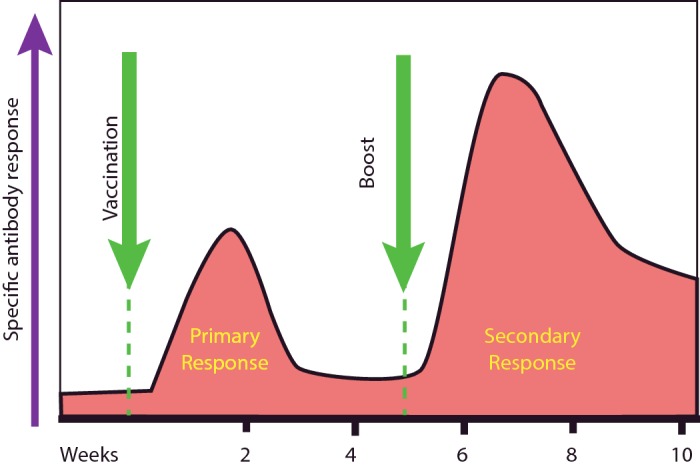
Antibody responses to vaccination Following the first immunization, increases in specific antibody can be detected. The levels of this fall but do not return to baseline. Following the boost, the secondary response is greater and the baseline is higher. Kinetics and levels vary between individuals and between different vaccines.

The classical measurement of immune memory focuses on the antibody response. Following an infection (or a vaccination), specific antibody levels rise, with a lag of about a week, to a peak around 10 days, and then fall off with time to lower levels that are not as low as in the naive state (Figure 8). Re-challenge with the same stimulus produces a faster (1–3 days), larger (more antibody is produced) and higher-avidity (as a result of the evolution and selection of higher affinity antibodies; Figure 7) response. Other changes are also apparent, particularly that the predominant isotype of the antibody response will usually have shifted to IgG (and sometimes IgA or IgE).

These well-documented and robustly replicable responses are clearly a necessary part of immune memory, but they are not the whole story. In parallel with changes that are occurring in the antibody response and can be measured in the serum, other important developments occur in different populations of antigen-specific lymphocytes.

B-cells develop into two kinds of immune memory cell. **Plasma cells** localize in bone marrow or mucosal tissues and produce high-affinity antibodies, for many years. Other B-cells become memory cells which do not actively secrete antibody, but remain in the circulation and the mucosal tissue long-term, retaining the potential to develop antibody production. By the time an individual becomes an adult, their blood contains antibodies produced from cells that have responded to many different infections.

T-cell populations are also shaped by an individual's history. The simplest change that can be measured experimentally is that, following an infection or an immunization, there is an increase in the fraction of the T-cell population with TCRs that recognize that infection. A simple interpretation of how this is ‘memory’ is that a higher frequency of specific cells means a shorter time between a new infection occurring and it being recognized. T-cells also undergo changes in their internal requirements for activation. Cells that have ‘seen’ an antigen are easier to activate. They have a lower threshold for triggering (responding to antigens at lower concentrations than naive cells) and become activated by signals that naive cells ignore. Their life expectancy is also extended, so they hang around longer. Furthermore, some subpopulations of T-cells home to the tissues where the infection was first detected. For example, if a pathogen enters through the skin, lymphocytes will take up residence here and remain, as though ‘on guard’. Although immunologists have been slow to enumerate these components of the memory response, it is becoming clear that a great deal of the immune resource is devoted to seeding organs with memory cells, particularly when these tissues are close to the outside world.

All of the changes discussed above seem to occur in a fashion that is independent of whether the offending infection is still present. But there are still questions here that are hard to answer, such as how serial infections, with different organisms, are handled. Is there an elastic capacity to keep all memory cells or do new memory cells drive out old? We know that certain infections regularly induce life-long protection and other infections do not. Understanding whether we can convert the latter into the former in some way is an important part of vaccine research.

In spite of all the different mechanisms that the immune response deploys, there are infections that regularly succeed in establishing long-term colonization. In this situation, can the immune response do anymore? Chronic infections, and chronic immune responses generally, can induce the immune system to take a further step. This is the local development of specialized immune tissue. It produces what is effectively a lymph node at the chronically inflamed site, which attracts dedicated B-cells and T-cells, and forms a structure that incorporates modified blood vessels and support tissues to increase the efficiency of local immune responses. However, in some conditions, such as autoimmune disease, these changes are associated with a poor outcome for the patient, because they represent a stronger underlying immune drive that does more damage to healthy tissue.

Immune responses are not just concerned with driving immunity forward; there are many negative-feedback loops that shut responses down. **Humoral** responses, mediated by antibody, use a specific type of receptor (a special type of **Fc** receptor) that senses the levels of Ig in the environment and shuts down its production once it reaches a certain level. T regulatory cells limit exuberant immune responses to self-antigens.

In summary, although the importance of immune memory is unquestionable, its implementation is a subtle interplay of cell types, effector molecules and timing at least as complex as the acute immune response and equally fascinating.

## Diseases linked to the immune system

### Immunodeficiency

One argument for the importance of immune systems, i.e. that every living thing devotes precious resources to them, does not address how crucial this is at different stages of life. Furthermore, animals and people with crippled immune systems grow, develop and reproduce fairly normally, if they are protected from pathogenic infections, so there is no intrinsic need for a functional immune system for life. This raises a question of how fundamental to good health the immune response is? Experiments of Nature have given us a very clear answer.

Rarely, but regularly, individuals are born without an effective immune system. This arises when uncommon mutations prevent immune cells from maturing. Such children have a very limited life expectancy. Without immunity, they are repeatedly attacked by the organisms that afflict all of us. For individuals with severe immunodeficiency, a successful bone marrow transplant is a standard life-saving treatment, using donor cells that do not harbour the dangerous mutation, or their own cells modified by genetic engineering to correct the defect.

Less dangerous, but still severe, are mutations that cripple a particular arm of the immune response. Patients whose complement proteins do not work suffer from repeated infections with bacteria that cause abscesses and pneumonia; patients with deficiencies in their natural killer cells are highly susceptible to herpesvirus infections. Patients who have macrophages that cannot digest the bacteria that they eat, develop recurrent abscesses that are difficult to treat. The most common are defects that arise in antibody-producing cells, and these conditions are often X-linked, i.e. encoded on the female X chromosome and so commoner in men than women. Clearly, in the wild, adequate immunity is crucial to a healthy childhood.

What about adults; does the ability to mount an immune response continue to be important as we age? Here, again, the answer is delivered through our study of disease. **HIV** infection cripples the immune system, producing profoundly immunocompromised individuals. Before the development of effective anti-retroviral therapy, HIV that reached the stage of AIDS was a death sentence. This was because patients developed uncommon infections and cancers, which could no longer be tackled by the ravaged immune system that had been destroyed by HIV.

**Immunosuppression** for other reasons is becoming increasingly common, because it is a useful treatment for many different diseases. Immunosuppression may be profound when patients have received transplanted organs or bone marrow. Sometimes it is more focused, for example targeting specific cytokines such as **tumour necrosis factor** (**TNF**) and interleukin (**IL**)**-17** or targeting molecules that regulate how immune cells migrate around the body. For example, drugs used to treat multiple sclerosis, which inhibit T-cell trafficking into the brain, are effective in reducing autoimmune disease, but occasionally lead to the re-emergence of previously suppressed infection, which was being held in check. Therapies that neutralize TNF give great relief to patients with rheumatoid arthritis, but, in some cases, this promotes the re-emergence of tuberculosis. These immunodeficiencies, arising because of infection or therapy in adults, indicate that an active immune response remains an important guardian of health throughout life.

### Autoinflammation

Defects in innate immunity, in complement pathways, which presented with recurrent infection, were identified in the 1970s. More recently, we have come to understand that mutations in the sensing systems that the innate immune system uses to calibrate danger, can lead to inherited illness. One important sensor is called the **inflammasome**, an intracytoplasmic structure that assembles from several proteins when the cell detects danger. Subunits of the inflammasome come together and activate an enzyme that then releases inflammation promoting cytokines. Once in the circulation, these cytokines can cause sickness and fever.

Inflammasome activation is triggered by changes in the environment that cause the subunits to assemble. The disease gout, which follows the deposition of crystals in small joints such as the toe, depends on inflammasome activation by these crystals, which lead to inflammation. Inherited diseases arise as a result of **gain-of-function mutations** in part of the inflammasome machinery that leads to spontaneous assembly, or assembly following mild environmental change such as exposure to cold. Some of these mutations are compatible with a relatively normal life expectancy, and have a 50:50 chance of being passed on to the affected parent's offspring. Other related diseases are much more severe. And, although the underlying cause may be a mutation in a molecular pathway that is part of the innate immune system, large-scale and persistent inflammation can, in time, lead to the development of autoimmunity too.

### Autoimmunity

Autoimmune disease occurs when the adaptive immune system mounts an attack against healthy tissue. Recent research indicates that resistance to infection and susceptibility to autoimmune disease are intimately connected. Individuals with a ‘stronger’ immune system, which reacts aggressively to infection, are also more likely to develop autoimmune disease. This is another example of the delicate balance the immune system strikes between its response to the safe or the dangerous environment.

As with all other adaptive immune discrimination, the antigen that is targeted determines that pattern of disease. This means that some autoimmune diseases affect a number of different organs [e.g. systemic lupus erythaematosus (SLE), where the targets are common components of nucleus and cytoplasm components], whereas others attack a single organ, for example the brain in the disease multiple sclerosis (where the targets are proteins found only in the brain). Early theories of autoimmunity postulated that it occurred because of the escape from the thymus of aberrant T-cells that could see autoantigens. But it has been well established that all individuals harbour potentially dangerous T-cells and the key question is not how they escape from the thymus, but how they become activated to a state that causes disease.

There are two situations in which this has been shown to happen. The first follows a sudden change in a tissue. This might be an infection that kills cells or an accident that causes local damage. This tissue damage releases signals that trigger an immune response against itself. This would usually be contained by feedback inhibition, but, when it is not, autoimmune disease can follow. The second is when antigens derived from the tissue and those produced by an infection are very similar and the host has a TCR that can recognize both. If the T-cell is activated and expands, discrimination between the infection and the tissue fails and the normal tissue antigens provoke an ongoing immune response. Both of these processes can be demonstrated in animal models of autoimmune disease.

However, in human disease, we rarely know by which of these pathways disease has developed. Infections that contain cross-reactive antigens probably also always cause tissue damage, and an accident that is bad enough to damage tissue is often accompanied by broken skin and the possibility of infection. Triggering events are also often clinically trivial. When patients receive their first diagnosis of autoimmune disease, they often have a history of recent illness. But it is unusual that they have been sick enough to have seen a doctor. So there is no connection between the seriousness of the triggering event and the severity of the autoimmune disease that follows.

**Figure 9:**
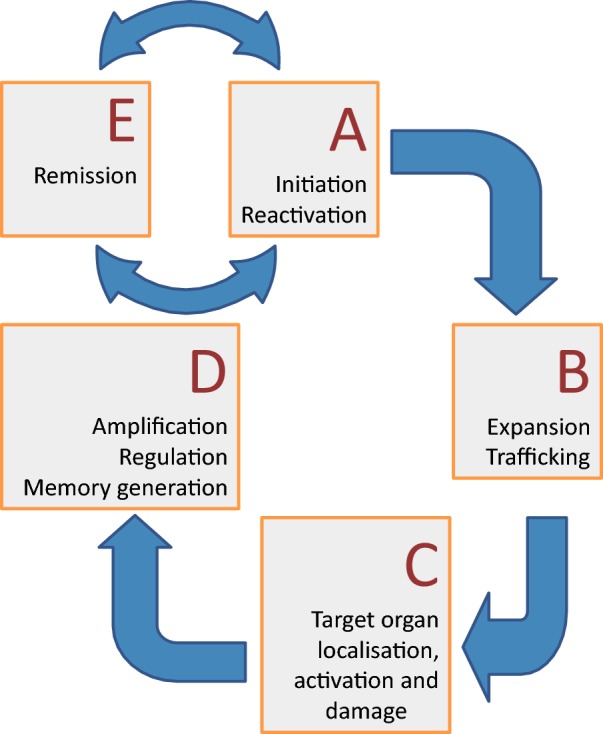
Overview of autoimmunity An autoimmune episode starts (**A**) with the initiation or reactivation of an immune attack directed against self. The T-cells expand and traffic (**B**) to the site where the antigen is found, are activated and damage the tissue (**C**). With time, the response is regulated and subsides, although the immune memory remains (**D**). Future infection may then trigger a relapse of the remission (**E**).

After an autoimmune has been diagnosed, more often than not, it becomes a chronic problem. It is also likely to wax and wane. This ‘relapsing and remitting’ pattern reflects underlying waves of activation and regulation, changing the level of autoimmune attack (Figure 9).

Autoimmune diseases invoke immune responses that are as complex as those for infection, but it is useful to separate them into situations where the tissue is attacked by a destructive cell-based inflammation and those where the pathology comes from attack by antibodies. Cell-mediated attacks on a tissue are principally destructive. Type 1 diabetes, in which every single insulin-producing cell in the pancreas is wiped out, and multiple sclerosis, in which inflammatory lesions in the central nervous system destroy tissues, are examples of this. Antibodies can also initiate tissue destruction, but they sometimes have a second type of effect if they target a cell-surface receptor. In this situation, they can produce inappropriate triggering within the target cell or block normal function. In autoimmune disease targeting the thyroid gland, antibodies sometimes bind to the **thyrotropin receptor** causing the release of hormones that drive up the metabolic rate of the patient. In **myasthenia gravis**, antibodies target the acetylcholine receptor, interfering with signalling to muscles leading to profound weakness. Sometimes autoantibodies attack the immune response itself, for example blocking the action of the cytokine IL-17.

The treatment of these conditions is two-pronged. If possible, replacement of missing hormones such as insulin is instituted. And if the disease is destructive, the immune system can be suppressed. Unfortunately, current therapies are non-specific and lead to various degrees of immunosuppression which has its own dangers. Every patient represents a difficult choice between the risk of the treatment and its benefit. Research in animals has demonstrated that it is possible to produce antigen-specific immunosuppression, but this has not yet been applied successfully in autoimmune diseases in patients.

### Allergy

On the other hand, antigen-specific therapies have proved to be useful in allergy. Like autoimmune disease, allergy is caused by an inappropriate immune response. In this case, however, the triggering antigen is not an autoantigen, but a usually innocuous environmental protein. Allergic responses can arise to a very broad range of different stimuli. These might be natural, such as the pollens that cause hay fever, or they may be drugs, such as the antibiotic penicillin. Allergic responses are very specific, as you would expect, because they are adaptive immune responses. Every individual's hay fever is triggered by one type of pollen, not by any pollen. Patients with an allergy to penicillin are safe taking other kinds of antibiotic.

Allergies are the commonest type of acquired immune disease. They usually depend on an IgE-mediated immune response to the environmental antigen. As with triggering autoimmune disease, the initial sensitizing event may not even be perceived, but it leads to high-affinity IgE antibodies being generated. The IgE binds to granulocyte cells and acts as a receptor for the allergen, releasing powerful inflammatory mediators when they are triggered. Such cells are abundant at mucosal surfaces where it is thought that their normal role is to eliminate dangerous toxins, which explains the sometimes rapid and dramatic symptoms that their activation produces.

Allergies have been successfully treated for many years by a technique called desensitization: starting from tiny doses, a patient is gradually exposed to increasing amounts of the triggering antigen. This gradual escalation stimulates negative-feedback mechanisms that keep the allergic reaction in check. For patients with severe reactions, this treatment will protect them from a potentially fatal reaction if they encounter the allergen in the environment.

Both allergy and autoimmune disease are becoming much more common in first-world populations. Studies have consistently shown that the fraction of people developing these diseases is increasing. Why this should be the case has not been proved, but one suggestion, the ‘hygiene hypothesis’, postulates that modern standards of public health, which have had a profound impact on the timing and type of infections that children encounter as they grow up, have changed the overall balance of the immune response. Childhood mortality is much lower because of better public health, but immune systems that develop in this cleaner world may be less educated and more prone to errors in discrimination. Such ideas are difficult to prove conclusively, but have received partial support in numerous experiments and epidemiological investigations that highlight the subtle and complicated interplay between an individual immune system and the environment.

## Applications of immunology

### Transfusion and transplantation

Sophisticated theories of blood circulation, which became prominent in the 17th Century, informed experiments transferring blood, for example from one person to another or from animals to humans. Sometimes these treatments were described as successful, but often they were fatal, especially if repeated a second or third time, which we now know would have allowed enough time for immunological memory to develop.

Investigating the source of this memory, in 1903 Karl Landsteiner reported that human serum contained antibodies that targeted red blood cells for destruction. The rules governing red blood cell immunity were relatively simple. Individuals had destructive antibodies against any red cell antigen they did not express themselves. The first antigens to be described were called A and B. A patient with ‘A’ antigens has antibodies that destroy red blood cells expressing ‘B’ antigens and vice versa. Red blood cells that did not express either antigen (blood group O) could be transfused into any recipient (Table 3). Understanding these patterns of response set the scene for the development of safe blood transfusion.

**Table 3.** Blood group incompatibilities

Blood group	Serum contains antibodies against	Serum lacks antibodies against
A	B	A and O
B	A	B and O
O	A and B	O
AB	Nil	O, A and B

Transplanting whole tissues was a much more difficult challenge. Ensuring that blood groups matched was not enough to prevent rejection. In 1944, it was not even generally accepted that rejection involved an immune response. Peter Medawar, working on skin grafting in rabbits, demonstrated that this was the case by establishing that second grafts were rejected more rapidly than first grafts. He then went on to estimate a minimum number of antigens that was necessary to explain the fact that no rabbit accepted a graft from any other. He showed that the fewest number of antigens that could produce this result was seven, a finding he described as “astonishing”. Although there were three major blood groups, he concluded that, for skin transplantation groups, there must be at least 127. Although as we now know, because this process is regulated by thousands of different MHC alleles, this was a large underestimate, it set the scene for scientists and surgeons to understand that successful transplantation strategies must always incorporate a plan to manage the immune system. Since then tissue typing has played a key role in this process, first using methods detecting antigen expression on white blood cells and more recently by using DNA sequencing to detect HLA genes to find well-matched organ donors.

### Learning to exploit antibodies

The specificity of the immune response stood out as one of its key features from the earliest immunological studies. Exploiting this specificity moved immunology rapidly from a purely experimental science into practical applications such as specific treatments for disease and new methods to interrogate the complex chemical make-up of life. At first, these techniques often depended on producing antibodies in animals by immunization, harvesting their serum and preparing them to be used as drugs. This type of therapy provided **passive immunity**.

This may sound old-fashioned in the modern age of molecular medicine, but in fact the use of antisera remains important to this day. If you are unlucky enough to be bitten by a venomous creature in Australia, you may need to be treated with ‘antivenom’. These are produced by injecting horses repeatedly with innocuous amounts of snake or spider venom, collected from reptiles or arachnids kept in the Australian Reptile Park. The horses develop neutralizing antibodies that can be purified from their blood and used in treatment. The horse antibodies, given to the victim, bind to the venom, neutralizes its toxic effects and dramatically reduce the mortality associated with snake or spider bite. Antivenom treatment is administered somewhere in Australia on most days of the year.

Antivenoms are mixtures of antibodies with many different specificities and affinities. Our ability to produce pure antibody reagents with defined specificities took an enormous leap forward in 1975 when César Milstein and Georges Köhler published a report describing a method to produce a clonal population of antibody-secreting cells. The research problem they were interested in was tracing the biochemical changes in antibody structure that occurred as the antibody response matured, and they needed large amounts of identical antibody protein to do this. The technique they developed was immediately applied by others. For example, immunizing animals with cells purified from the immune system produced antibodies that bound to different proteins on the surface of **leucocytes**. These quickly became crucial tools in investigating the function of these proteins in the immune response.

**Figure 10:**
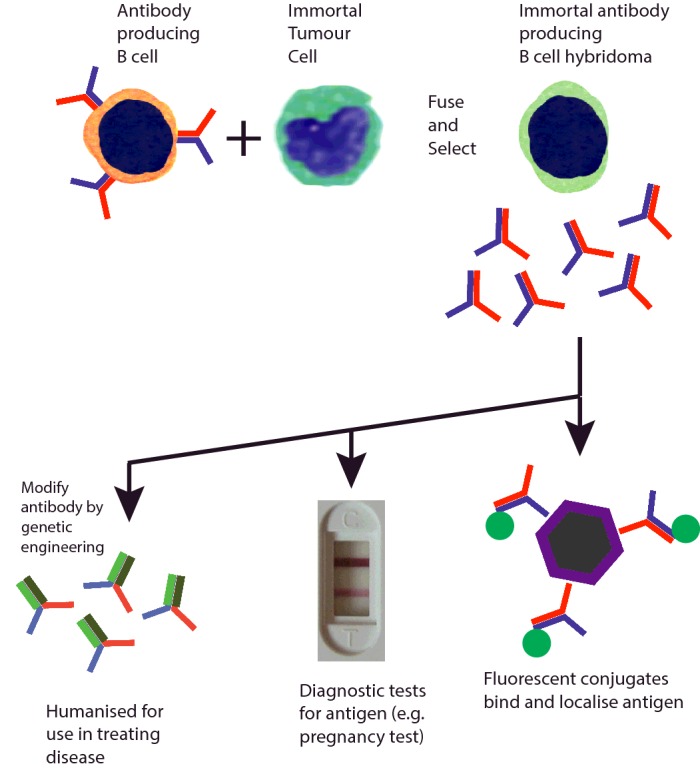
Monoclonal antibody production and application Immunization leads to B-cells producing antibodies that bind to an antigen of interest. Individual B-cells are fused with partners that immortalize them and the resulting B-cell hybridomas produce identical (monoclonal) antibodies indefinitely. Following screening, relevant hybridomas are selected and expanded to produce large quantities of uniform antibody-producing cells. Such antibodies have many applications, for example locating antigen in tissue samples, providing the basis of a specific test for pregnancy hormone or being genetically engineered to use in immunotherapy of disease. Pregnancy test: Nabokov, en.wikipedia.org/w/index.php?curid=36047325

The technique of monoclonal antibody production is carried out in two parts: first, an immunization/cell fusion step and, secondly, a selection/screening step (Figure 10). The goal is to immortalize cells producing antibody. Immunization generates donor B-cells that are antigen-specific. These cells are fused with a B-cell lineage tumour cell, that can grow indefinitely, but which has a mutation that means that the tumour dies in the presence of a drug called aminopterin. When the B-cells fuse, they become a hybrid of the two donors, and their genes mix together. The antigen-specific B-cell provides the genes that code for the antibody and enzymes that allow the hybrid to survive in the presence of aminopterin. After fusion, all of the cells are grown in medium containing aminopterin and only cells that have successfully incorporated genes from the donor B-cells survive. All of the immortal cells that come from this process have fused with antibody-producing cells and those with the greatest specificity can be identified by screening. The immortal cells are called **hybridomas** and they produce **monoclonal antibodies**.

Harnessing this antigen-recognizing ability of the immune system led to an explosive growth in research applications, medical diagnostics and therapies for diseases from arthritis to cancer. These approaches continue to be developed and their usefulness extended. Monoclonal antibodies are used in research and diagnostic laboratories all over the world to probe for the presence of specific molecules in clinical samples, on the surface of cells prepared from the blood or in tissues obtained by biopsy. They have provided a bedrock technology for simple to use clinical tests such as for pregnancy or viral infections. These tests rely on the ability of antibody to bind one specific molecule among a mixture of thousands. This means that they can work with a drop of blood or a sample of urine, without requiring complicated biochemical preparation.

Antibodies for treating human disease often benefit from further genetic engineering. This is because antibodies from non-human species will provoke an immune response. To use them as drugs, it is desirable to alter the structure of the antibody so that it has a human protein scaffold, for example replacing a rat Fc tail with a human Fc tail. Their use in immunotherapy is discussed below.

### Vaccination

In 2014, the Centers for Disease Control and Prevention in the U.S.A. published an estimate of the impact of their national vaccination programme over the previous 20 years. They concluded that over the lifetime of the immunized individuals that there would be 21 million fewer hospitalizations and 732000 fewer deaths. The success of vaccination is rarely emphasized in media coverage, but it is an enormous and cost-effective benefit. Vaccination, as a public health strategy to harness the immune response, pre-dates by at least a century acceptance of the germ theory of disease, which provides a mechanistic understanding of why it is effective. It has a crucial and continuing role in the fight against both ancient and emerging infections.

The most notorious disease against which vaccination has been applied is smallpox. A scourge among humans since at least 1100 years BCE, it was a terrifying condition, transmitted through the air, which killed 20–30% of individuals who became infected and left the survivors severely disfigured by scarring. Among naive populations, i.e. those which had never encountered the infection, it was even more deadly. In 1738, smallpox killed half of all the Cherokee Indians in the U.S.A. Vaccination against smallpox was pioneered by Edward Jenner in the 18th Century, and in 1958, the World Health Organization (WHO) adopted a resolution to attempt global eradication. The disease was cornered by a combination of vaccination, surveillance and containment of epidemic hotspots. Factors that helped successful eradication included that humans played an essential role in the life cycle of the virus, that there was no natural reservoir in the animal kingdom, and that smallpox did not establish a carrier state, in which symptomless individuals were contagious. As progress reducing smallpox outbreaks continued and the number of victims fell, the WHO optimized a strategy of tracing and vaccinating contacts, working outwards from the patient to contain the disease. Through the dedicated work of many individuals, the world was certified free of the smallpox in 1979. This great success has been challenging to replicate with other diseases. To date, the only other infection to be exterminated entirely from the wild by vaccination is rinderpest. This was caused by a virus related to measles which affected cattle, and had a mortality approaching 100% in non-immune populations.

Local eradication of other diseases through vaccination has proved possible. Polio has also been eradicated in almost all countries, thanks to vaccination. Following a programme in Finland, starting in 1982, measles, mumps and rubella were eliminated from the indigenous population between 1996 and 1997. Maintaining this freedom from disease remains an ongoing challenge; as the immunity of the population wanes and the memory of how dangerous these illnesses were fades, uptake of vaccination falls, but these viruses continue to circulate in other countries, from which they could be reintroduced to a now susceptible population.

Vaccination works by immunizing individuals with a ‘safe’ version of the infectious agent, to educate the immune system without causing severe illness. Some vaccines, for example that for polio, use weakened (attenuated) strains of live virus. For other conditions, heat-killed preparations may be preferred or the vaccine may contain recombinant proteins that have been synthesized and purified from cell culture (e.g. hepatitis and human papillomavirus vaccines). Vaccines are remarkably safe, but each new vaccine is unique and occasional unanticipated complications do occur. Where these are common, they will be detected early in clinical trials, but if they are rare they will only become evident after millions of doses of the vaccine have been given. These rare outcomes are impossible to predict and must be identified by monitoring the vaccinated. Such complications do less damage than the diseases that they are a protection from when these infections are at large. But as the illnesses themselves become rarer, the benefits that vaccination produces become more difficult to appreciate.

Devising vaccines for new infections is not straightforward. It is instructive to set against the astonishing success that is the eradication of smallpox, the failure to date to discover a vaccine of similar efficacy that is effective against malaria or HIV. This does not reflect a lack of effort, but does illustrate that our ability to harness and manipulate the immune response remains limited. There is still much to learn.

### Immunotherapy

Immunotherapy is treatment that exploits our understanding the immune response in animals or people to achieve a medical benefit. It may use the specificity of the immune response or target general mechanisms such as co-stimulation, either enhancing or inhibiting them. The earliest example of effective immunotherapy is vaccination against smallpox, discussed above, but there are many other areas into which these techniques extend. One of the first clinical applications of immunotherapy was the development of passive immunity against diphtheria toxin, introduced by Emil Behring and based on work reported in 1890. Behring and Kitasato Sibasaburo immunized guinea pigs with attenuated toxin and discovered that their serum could cure animals infected with virulent strains of the disease. In 1891, this approach was used to save a child with diphtheria (at the time, this disease was killing more than 50000 children a year in Germany). By 1892, this antitoxin was being produced commercially, first in Germany and later in London. Such anti-toxins continue to have a role in modern therapy to this day.

In autoimmune diseases, the goal of immunotherapy is to shut off the immune response. The gold standard would be a treatment that selectively disables only the antigen-specific response directed at self, leaving the rest of the immune system intact to defend the patient against infection. This has proven to be extremely difficult to achieve in human populations, where immune responses are mediated through a myriad of overlapping antigen specificities, but it can be demonstrated in animal models of autoimmune disease, which provides hope that such therapies can be developed. The overarching idea is that it should be possible to reprogramme the immune response to ignore specific autoantigens, something that might be considered the reverse of immunization.

In the absence of effective autoantigen-specific therapies for autoimmune disease, other approaches have proved useful. One is to neutralize the soluble mediators that signal inflammation. This has been applied to rheumatoid arthritis, an autoimmune disease that attacks the joints. Blocking the cytokine **TNF** brings rapid and long-lasting benefit to patients. This is done in a number of ways, but one is a modern reinvention of Behring's approach. Mouse monoclonal antibodies that recognize and neutralize TNF were identified and then engineered so that the backbone of the antibody was human. These can then be injected into patients to neutralize the actions of the cytokine. Of course, TNF is not just important for mediating arthritis and patients who receive this treatment are immunosuppressed. Although this suppression is not as profound as it would be following an organ transplant, there is still an increased risk of reactivation of infections such as tuberculosis. Other anti-cytokine antibodies have also proved useful, for example they are used to treat autoinflammatory diseases (anti-**IL-1** and anti-**IL-6**) and skin diseases such as psoriasis (anti-IL-17).

As well as blocking soluble mediators, antibodies can block interactions between molecules on the surface of cells. This effect is used in a treatment that stops T-cells moving out of the blood and into the brain of patients with multiple sclerosis. This therapy developed from research in mice that showed that lymphocytes used specific receptors to regulate how they moved into different organs. The equivalent molecules are used by lymphocytes in humans and blocking them with antibody reduces the number of attacks of disease in patients with multiple sclerosis. A side effect of these drugs is that they limit immunosurveillance. In rare cases, this precipitates the emergence of dangerous infections in the brain, which were previously held in check by the immune system.

Cancer can also be treated by exploiting the immune response. One early strategy for successful immunotherapy for cancer used monoclonal antibodies that blocked receptors which the tumour cells used to receive growth-promoting signals. These treatments are beneficial when the cancer cells express these receptors, but their impact is generally of only a limited duration and is eventually followed by a relapse. This is because, similar to the way that viruses live many lifetimes to that of a human, a growing cancer is a fast-evolving threat to health. Cancers arise when mutations that permit unrestrained growth develop, and this growth is associated with much less reliable checking of genetic fidelity. Modern techniques have allowed researchers to develop family trees based on mutations in a cancer's genes, showing how they consist of related lineages of cells that peel off from the parental line and expand through time. Every mutation that develops is an opportunity for the immune system to push back, by recognizing tumour antigens on their surface in their MHC molecules. If this mechanism were failsafe, tumours would never progress beyond this stage. But cancer can escape the immune system by deploying a number of mechanisms that shut the immune responses down. Understanding how this occurs has stimulated the development of a number of new therapies that are having an astonishing impact against some types of cancer.

Two lines of research were important in reaching these conclusions. One came from studies that showed that a cancer, which evolved through time, in an environment where there was an intact immune response gradually developed the ability to grow despite immune system control. This suggested that the immune response kept the cancer in check for a while and exerted a pressure that selected mutations which permitted escape. The second strand came from workers interested in co-inhibitory cell-surface molecules regulating lymphocyte activation. It was discovered that these were an abundant family of different molecular receptors, which could limit immune activation or even shut it down completely. Cancers learn to use the signals that turn lymphocytes off, persuading them not to kill. But the tumours cannot eliminate the immune cells and so they remain in an uneasy truce with the tumour, present but not activated.

Once it was realized that some tumours were constantly delivering negative signals to immune cells, it became feasible to devise strategies to interfere with these signalling pathways. Once again, targeting them with antibodies to block this signalling was an obvious and appropriate approach. Drugs that do this have now been tested in patients and, in some cases, the results have been quite remarkable. As of 2015, two pathways have been successfully targeted in clinical trials, inhibiting molecules called CTLA4 and PD1. There is some evidence that attacking both together is even more effective than either used alone. Following treatment with these drugs, some patients thought to have weeks or months to live have developed long-term remissions that have been so striking that the possibility that some of them are cured is beginning to be considered. Such powerful interventions are not without side effects. Unleashing immune responses can be intolerable and sometimes fatal; patients who cannot control lymphocyte activation may develop autoimmune diseases, unrelated to their cancer, as a result of subclinical disease that was previously held in check. However, in cancer cases, the benefits often outweigh the side effects, and, as we learn more about how to apply such treatment, we will learn how to better manage the accompanying autoimmunity.

Cancer immunotherapy that uses killer cells to selectively target the tumour cells is also being tested in clinical trials. By combining genetic engineering with cell culture techniques, a number of small studies have established that it is possible to create T-cells that will target tumour cells and cannot be turned off. These cells [chimaeric antigen receptor (CAR) T-cells] are genetically engineered and grown outside that patient's body and then reinfused. On their return, they expand and destroy tumour cells. Although side effects due to extensive cytokine release are an important complication, these therapies too can lead to very long-term remissions. This proof of principle raises the possibility in the future of making an individual's unique cancer antigens the target of an immune response. The barriers to these types of treatment are many, not least their cost, but they are a very real opportunity for effective, immune-based and individually tailored cures for cancer.

## Conclusion

Immunity works in a co-ordinated fashion to respond to numerous threats from the environment. It is essential to good health, from the moment of conception, when the mother's immune system starts protecting the growing baby, until old age. As medicine has progressed, physicians have slowly learned how to apply an understanding of the fundamentals of immunology to reinforce and repurpose the immune response, providing greater protection against infection or targeting cancers. Immunotherapy has been improving human health since Edward Jenner coined the term vaccination and it will have much more to contribute in the future.

## Figures and Tables

**Figure:**
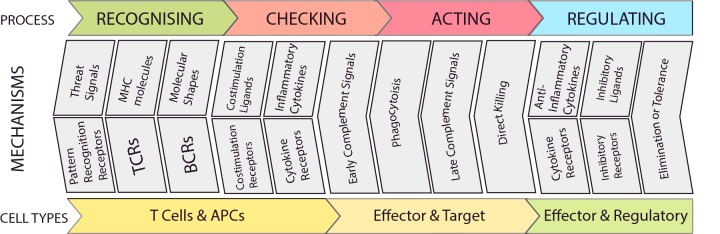
Overview of the Immune Process An immune response goes through a number of stages. It begins by recognising that there is a threat from an infection. The response is controlled and directed by a number of checking mechanisms that optimise the system in parallel with effector mechanisms that target, limit, damage or destroy the threat. To contain ongoing damage, regulatory mechanism can dampen responses, promote memory and establish a state of tolerance.

## Notes

This article is a reviewed, revised and updated version of the following ‘Biochemistry Across the School Curriculum’ (BASC) booklet: Davey, B. *Immunology*. For further information and to provide feedback on this or any other Biochemical Society education resource, please contact education@biochemistry.org. For further information on other Biochemical Society publications, please visit www.biochemistry.org/publications.

## Abbreviations

APC: antigen-presenting cell
BCR: B-cell receptor
HLA: human leucocyte antigen
Ig: immunoglobulin
IL: interleukin
MHC: major histocompatibility
PAMP: pathogen-associated molecular pattern
TCR: T-cell receptor
TLR: Toll-like receptor
TNF: tumour necrosis factor

## Appendix 2. Glossary of terms

Immunology, like other sciences, is encoded in a language that can seem a little impenetrable. It has its own dictionary which allows immunologists to concentrate paragraphs of meaning in single words. This helps us to play with different concepts in conversation, and saves time when we write about the science, but it raises a barrier to common sense comprehension. Sometimes this occurs because the concepts *are* genuinely difficult. But, more often, it is simply that we deal with a lot of unfamiliar things that need to be named. Thousands of individual molecules, dozens of different types of cell. To try to help readers to navigate this thicket, I have provided a glossary that can be used to translate aspects of the text.

**Affinity** The strength of binding between two biomolecules.
**Affinity maturation** The process by which sequential rounds of mutation and selection increase the affinity with which antibodies bind free antigen.
**Alleles** Different structural variants of the same gene; produces slightly different versions of a protein.
**Amino acid** Fundamental building block of protein. Our genes can code for 20 amino acids.
**Antibodies** Soluble proteins, produced from B-cells by rearrangement of their B-cell receptor. Antibodies bind to their targets with very high affinity and therefore specificity.
**Antigen** A structure recognized by the adaptive immune system.
**Antigen-presenting cell (APC)** A cell that presents antigen to T-cells.
**Apoptosis** Process of cell suicide. Carried out by a proteolytic cascade.
**Autoimmune disease** Disease caused by the immune system attacking normal tissues as if they were infected.
**Autoinflammatory** Disease state that arises through the uncontrolled activation of the innate immune system.
**Autoreactive** Reactivity to a component of the self.
**B-cell** Lymphocyte that develops in the bone marrow (hence B) and then moves to secondary lymphoid tissue, where it may become an antibody-producing cell.
**B-cell receptor (BCR)** The receptor on B-cells that binds free antigen. Incorporated into secreted antibodies that therefore have the same binding specificity as the BCR.
**Basophils** Granulocyte cells that are stained by basic dyes.
**Biologics** Complex molecular structures (such as antibodies) adapted to use as drugs.
**Bone marrow** Found inside bones. In adults, most cells of the immune system start life in the bone marrow, going through a number of different stages before they are released into the blood.
**Caspases** Family of enzymes that digest proteins.
**CD4** Cell-surface protein marking T-helper cells.
**CD8** Cell-surface protein marking cytotoxic T-cells.
**Cell membranes** Cell membranes contain the internal cellular environment. They control signals in and out of the cell. Many specific structures (receptors) that span the membrane are devoted to these processes.
**Clotting** Complex biochemical process that arrests the flow of blood in the event of vessel injury.
**Complement** Part of the innate immune response, a family of proteins that amplify immune responses and kill bacteria.
**Co-stimulation/co-inhibition** Signals that modulate the response of lymphocytes triggered through their antigen-recognizing receptor.
**Cytokines** Cytokines are a family of ligands (usually soluble), produced from one cell and signalling to another. The information that they provide to receiving cells regulates growth and effector function.
**Cytoplasm** The internal environment of the cell.
**Cytotoxic** Cell killing.
**DNA** Deoxyribonucleic acid. Used to store the genetic code in the nucleus.
**Effector function** A process by which cells alter the environment around them.
**Enzymes** Proteins that work as catalysts, speeding up chemical reactions without themselves being altered.
**Eosinophils** Granulocyte cells that are stained by acidic dyes.
***Escherichia coli*** Bacterium found in the normal microbiome and widely used in molecular biology.
**Fab fragment** The head of the antibody containing the antigen recognising parts of the immunoglobulin.
**Fas/FasL** Receptor ligand pair that induces cell suicide (apoptosis).
**Fc fragment** The tail of an antibody. It defines how the antibody functions.
**Gain-of-function mutations** A usually dominant mutation that exaggerates the normal activity of the affected gene, causing a new cellular phenotype.
**Gene** The genetic instructions that specify the amino acid sequence of an individual protein.
**Granulocytes** White blood cells containing granules of chemicals such as histamines that can be released rapidly on stimulation.
**Granzyme** Enzyme that induces cell suicide (apoptosis).
**Histamines** Soluble inducer of an inflammatory response. Derived from the amino acid histidine.
**HIV** Human immunodeficiency virus. Produces the infection that causes AIDS.
**Human leucocyte antigen (HLA)** Alternative name for MHC in humans. Matching is important for organ transplantation.
**Humoral** Referring to antibody/B-cell-mediated immunity.
**Hybridomas** Immune cells immortalised by fusion with a tumorous cell line, which provides self-replicating source of cells that, for example, secreted a singly type of antibody.
**IL-1, IL-6, IL-17** Cytokines that cause inflammation.
**Immunoglobulin (Ig)** Antibody proteins found in tissue fluids and the circulation.
**Immunoglobulin superfamily** Large family of proteins related structurally by containing one or more Ig domains or folds.
**Immunosuppression** A reduction in immune responses. Associated with an increased risk of infection.
**Inflammasome** Cytoplasmic complex whose assembly stimulates the release of inflammatory cytokines.
**Innate immune system** Immune system using receptors that recognize early signs of infection and respond rapidly to limit spread and promote adaptive immune responses.
**Interferon γ** A cytokine that stimulates macrophage activation.
**Isotype** Types of antibody; defined by the antibody tail (the Fc fragment).
**Leucocytes** White blood cells.
**Leukotrienes** Soluble inducers of an inflammatory response.
**Ligands** Ligands are the partners of receptors. They bind specifically, commonly initiating the transmission of signals across cell membranes. They can be soluble or bound to the cell surface.
**Lymph node** Specialized structure that co-ordinates interactions between APCs, T-cells and B-cells. They form a widely distributed network throughout the body, close to sites of potential antigen entry.
**Lymphocytes** Immune cells that provide adaptive immunity. T-lymphocytes recognize antigen processed from infections; B-lymphocytes produce antibodies that bind free antigens.
**Lysosome** Intracellular organelle containing proteolytic (protein-cutting) enzymes.
**Lysozyme** Enzyme that damages bacterial cell walls.
**Macrophages** White blood cells that engulf and digest invading micro-organisms.
**Major histocompatibility (MHC) determinants** Polymorphic molecules that bind peptides processed from proteins. Class I (MHC I) binds peptides typically eight or nine amino acids long, class II (MHC II) accommodates peptides of ten to 30 residues).
**Measles virus** The RNA virus that causes measles.
**Microbiome** The microbial ecology that normal healthy individuals carry with them.
**Monoclonal antibodies** Antibodies of a single specificity secreted in large amounts by hybridomas.
**Myasthenia gravis** Autoimmune disease that leads to weakness because of the presence of antibodies that inhibit normal muscle function.
**Natural killer cells** Innate immune system cells that kill cells infected by virus or transformed into cancers.
**Neutrophils** White blood cells that fight infection by phagocytosis and by releasing sterilizing chemicals that kill invading micro-organisms.
**Nucleic acid** Chemical used in genes.
**Nucleus** Cell organelle containing the genetic code (DNA).
**Opsonization** Process by which antibodies coat a structure, to stimulate phagocytosis.
**Passive immunity** The transfer of immune function by transfusion of specific anti-sera, for example anti-venoms from horse serum.
**Pathogen** A living organism that causes disease.
**Peptides** Short stretches of amino acids.
**Perforins** Secreted proteins, released from cytotoxic cells, which produce holes in cell membranes.
**Plasma cells** Antibody-secreting B-cells.
**Phagocytosis** The process by which a cell engulfs material in its local environment.
**Phagosome** A membrane-bound structure inside a cell created by phagocytosis.
**Proteolytic cascade** A sequence of enzymes that activate each other, allowing a rapidly amplification in response. Widely used, for example in blood clotting, activation of complement and apoptosis.
**Protein** Protein molecules are made from chains of amino acids. They have a wide range of different functions.
**Rabies virus** The RNA virus that causes rabies. Symptomatic infection with rabies virus is almost always fatal.
**Receptors** Receptors bind specific ligands and produce signals that reflect the local concentration of the ligand at the cell surface.
**Rheumatoid arthritis** Autoimmune disease that attacks the small joints of the hands and feet.
**RNA** Ribonucleic acid. Genetic material used by all cells when producing proteins and by some viruses to store genetic information.
**Self-antigens** Antigens derived from normal proteins. Targeted in autoimmune disease.
**Sepsis** The clinical state that develops in severe systemic infection. Often life-threatening.
**Serum** Liquid component of blood, lacking cells and clotting factors, but containing antibodies and other soluble proteins.
**Spirochaete** Spiral-shaped bacterium. The pathogen that causes syphilis is a spirochaete.
**Spleen** Organ that co-ordinates interactions between APCs, T-cells and B-cells. Found in the abdomen. Also responsible for digesting red blood cells and recycling iron.
**T-cell** Lymphocyte that is programmed in the thymus (hence T) and which recognizes antigen bound to MHC molecules.
**T-cell receptor (TCR) complex** The receptor on T-cells that binds peptide MHC molecules, and signals into the cell.
**Thymus** The organ, found in the chest, in which T-cells bearing useful TCRs are selected.
**Thyrotropin receptor** Receptor on thyroid cells. Stimulation leads to the release of hormones that increase the metabolic rate.
**Tissue type** The tissue antigens (mainly MHC molecules) possessed by a single individual that are recognised by the immune system and which stimulate the rejection of transplants.
**Toll-like receptor (TLR)** Receptor belonging to the Toll-like family of proteins first described in insects. One of many families of receptor that activate the innate immune system.
**Toxins** Poisons.
**Tumour necrosis factor (TNF)** Cytokine released by macrophages that stimulates immune cell effector functions that promote inflammation.
**Vaccination** The deliberate recruitment of the adaptive immune response to develop memory of a specific infection in a therapeutic setting.
**Viruses** The smallest organisms, viruses need to co-opt the protein producing machinery of larger cells to reproduce.

## Appendix 3. A note on scale

The microscopic world spans a wide range of sizes. A cell is much larger than a molecule. To try to help express this, I have drawn a ruler, and marked on it where some relevant structures lie. It is important to realize that the scale I have chosen increases by a factor of 10 between each tick mark (it is logarithmic, not linear), meaning that the lymphocyte is 10000 times wider that the amino acid tryptophan (not five times, which would be the case if this was a linear scale). Note also that in the real world, where volume is usually more relevant than width, the cell is approximately 10^9^ times bigger than the individual proteins that give it its identity.

Picture acknowledgements. Tryptophan: this file is made available under the Creative Commons CC0 1.0 Universal Public Domain Dedication. Lysozyme: Jawahar Swaminathan and MSD staff at the European Bioinformatics Institute https://commons.wikimedia.org/w/index.php?curid=6292018; SV40 virus: this file is licensed under the Creative Commons Attribution-Share Alike 3.0 Unported license. *E. coli*: Janice Haney Carr; Usage: public domain (CC0).
